# Privacy-preserving genome-wide association studies on cloud environment using fully homomorphic encryption

**DOI:** 10.1186/1472-6947-15-S5-S1

**Published:** 2015-12-21

**Authors:** Wen-Jie Lu, Yoshiji Yamada, Jun Sakuma

**Affiliations:** 1 Graduate School of Systems and Information Engineering, University of Tsukuba, Ten'nodai 1-1-1, Tsukuba, Japan; 2 JST CREST, Honchou 4-1-8, Kawaguchi, Japan; 3 Life Science Research Center, Mie University, Kurimamachiya-cho 1577, Tsu, Japan

**Keywords:** GWAS, Outsourcing, Fully homomorphic encryption

## Abstract

**Objective:**

Developed sequencing techniques are yielding large-scale genomic data at low cost. A genome-wide association study (GWAS) targeting genetic variations that are significantly associated with a particular disease offers great potential for medical improvement. However, subjects who volunteer their genomic data expose themselves to the risk of privacy invasion; these privacy concerns prevent efficient genomic data sharing. Our goal is to presents a cryptographic solution to this problem.

**Methods:**

To maintain the privacy of subjects, we propose encryption of all genotype and phenotype data. To allow the cloud to perform meaningful computation in relation to the encrypted data, we use a fully homomorphic encryption scheme. Noting that we can evaluate typical statistics for GWAS from a frequency table, our solution evaluates frequency tables with encrypted genomic and clinical data as input. We propose to use a packing technique for efficient evaluation of these frequency tables.

**Results:**

Our solution supports evaluation of the *D′ *measure of linkage disequilibrium, the Hardy-Weinberg Equilibrium, the *χ*^2 ^test, etc. In this paper, we take *χ*^2 ^test and linkage disequilibrium as examples and demonstrate how we can conduct these algorithms securely and efficiently in an outsourcing setting. We demonstrate with experimentation that secure outsourcing computation of one *χ*^2 ^test with 10, 000 subjects requires about 35 ms and evaluation of one linkage disequilibrium with 10, 000 subjects requires about 80 ms.

**Conclusions:**

With appropriate encoding and packing technique, cryptographic solutions based on fully homomorphic encryption for secure computations of GWAS can be practical.

## Introduction

Because of recent advances in DNA sequencing technologies, the cost of DNA sequencers is dropping rapidly. As a result, the scale of genomic data used by researchers is becoming larger and larger. To conduct computations on a large-scale genomic dataset, a cloud server that provides computational resources at low cost is regarded as a promising option.

It is difficult to argue that genomic and clinical data are highly sensitive. Outsourcing these data to an external server raises concerns about the privacy of sensitive data. Consequently, for outsourcing of computation with genomic data, privacy should be rigorously preserved.

The fully homomorphic encryption (FHE) scheme is attracting attention as a tool for secure outsourcing of data analysis. FHE enables encryption of data and then carrying out arbitrary computation using the encrypted data without decrypting the data. The first FHE scheme was proposed by Gentry [1]: subsequent improvements [2,3] provided more practical FHE schemes.

Actually, FHE has been applied to secure outsourcing of computation that involves genomic and clinical data. Bos et al. [4] proposed a working implementation of cloud service for private computation of encrypted health data using FHE. Lauter et al. [5] demonstrated an approach to conducting private computation using encrypted genomic data with FHE. Unfortunately, these cryptographic solutions are not sufficiently time and space efficient to conduct a GWAS-scale computation, which can involve 300k SNPs for thousands or more subjects.

In this manuscript, we present a protocol for secure outsourced analysis of large-scale genomic data using FHE. Precisely, our proposed protocol evaluates a frequency table with encrypted genomic/clinical data as input. This enables us to outsource computation of typical statistics related to GWAS securely, such as the Hardy-Weinberg Equilibrium (HWE), *χ*^2 ^test for independence and Linkage Disequilibrium (LD). Our method works by virtue of the fact that we can pack integer vectors into a single ciphertext of a certain type of FHE. This packing technique enables us to evaluate a scalar product of integer vectors through a single homomorphic multiplication using the packing technique; such a batch style computation helps to conduct computation of GWAS-scale data in an efficient manner.

Our basic strategy is to compute allelic frequency tables and genotype frequency tables privately from encrypted genetic data. With these tables, GWAS-related statistics including *D′ *measure of LD, the Pearson Goodness-of-Fit, HWE, and the *χ*^2 ^test are conducted. In this work particularly, we apply our method to the *χ*^2 ^test and LD to demonstrate the effectiveness of our protocol.

We review an allelic frequency table and a genotype frequency table with two markers. Table 1 gives a view of a genomic dataset *D^g^*. Each record contains an explicit identifier ID and SNPs. Similarly, Table 2 gives a view of a phenotype dataset *D^p^*. Each record contains an explicit identifier ID*′ *to identify each subject and an attribute to indicate the disease status of the subject. Presuming that *M *subjects and *N *SNPs are involved, then the dataset *D^g ^*contains *N *rows, with each row containing *M *data points; the dataset *D^p ^*includes *M *rows.

**Table 1:** Raw genome data *D^g^*

ID	Genomic Data
1	CC CG CT GG AA
2	AG CT CT AG CT
3	CT GG CC AG AA
4	AA GG GG AG CC

**Table 2:** Raw phenotype data *D^p^*

ID'	Disease Status
1	Case
2	Control
3	Control
4	Case

Presuming that *A*, *a *are possible alleles. An allelic frequency table (Table 3) consists of 2 × 2 counts

**Table 3:** Observed allele frequency in a case-control study of *M *subjects.

	Allele Type		total
		
	A	a	
case	*o*_1_	*o*_2_	*N*_1_
control	*o*_3_	*o*_4_	*N*_2_
total	$N_{1}^{\prime }$	$N_{2}^{\prime }$	2*M*

$$\begin{array}{cc} o_{1}=2N_{\text{AA}}^{\text{case}}+N_{\text{Aa}}^{\text{case}} & o_{2}=2N_{\text{aa}}^{\text{case}}+N_{\text{Aa}}^{\text{case}}\\ o_{3}=2N_{\text{AA}}^{\text{control}}+N_{\text{Aa}}^{\text{control}} & o_{4}=2N_{\text{aa}}^{\text{control}}+N_{\text{Aa}}^{\text{control}} \end{array},$$

where $N_{\text{AA}}^{\text{case}}$ and $N_{\text{Aa}}^{\text{case}}$ are the observed population counts for genotype *AA *and *Aa *in the case group: $N_{\text{AA}}^{\text{control}}$ and $N_{\text{Aa}}^{\text{control}}$ are the observed counts for the control group.

A *χ*^2 ^test for the additive model is equivalent to the *χ*^2 ^test based on Table 3. The one degree of freedom (d.f.) test statistic is written as

$$\chi_{a}^{2}=\frac{2M{\left(o_{2}\left(o_{3}+o_{4}\right)-o_{4}\left(o_{1}+o_{2}\right)\right)}^{2}}{N_{1}N_{2}{N^{\prime }}_{1}{N^{\prime }}_{2}}.$$

In addition to a *χ*^2 ^test, we can evaluate the Hardy-Weinberg Equilibrium directly from an allelic frequency table similarly.

Given alleles (A/a and B/b) at two markers, a genotype frequency table (Table 4) with two markers is obtained that consists of 3 × 3 counts

**Table 4:** Genotype frequencies at markers *M*_1 _and *M*_2 _of *M *subjects.

		Marker *M*_1_			Total
			
		AA	Aa	aa	
	BB	*o*_11_	*o*_12_	*o*_13_	*N*_1_
Marker *M*_2_	Bb	*o*_21_	*o*_22_	*o*_23_	*N*_2_
	bb	*o*_31_	*o*_32_	*o*_33_	*N*_3_
	Total	$N_{1}^{\prime }$	$N_{2}^{\prime }$	$N_{3}^{\prime }$	2*M*

$$\begin{array}{ccc} o_{11}=N_{\text{AABB}} & o_{12}=N_{\text{AaBB}} & o_{13}=N_{\text{aaBB}}\\ o_{21}=N_{\text{AABb}} & o_{22}=N_{\text{AaBb}} & o_{23}=N_{\text{aaBb}}\\ o_{31}=N_{\text{AAbb}} & o_{32}=N_{\text{Aabb}} & o_{33}=N_{\text{aabb}}. \end{array}$$

The value $N_{ii^{\prime }jj^{\prime }}$ denotes the observed population counts for genotype i*i^' ^*and *jj^' ^*where $i,i^{\prime }\in \left\{A,a\right\}$, and $j,j^{\prime }\in \left\{B,b\right\}$.

We evaluate LD from Table 4. The linkage disequilibrium is calculated as *D *= p_AB _- p_A_p_B_, where probabilities p_AB_, p_A _and p_B _are computed, respectively, as (2*o*_11 _+ *o*_12 _+ *o*_21_)/2*M*, $\left(2N_{1}^{\prime }+N_{2}^{\prime }-o_{22}\right)/2M$ and (2*N*_1 _+ *N*_2 _− *o*_22_)/2*M*. We omit the frequency *o*_22 _to avoid the problem of haplotype ambiguity, especially when only genotypes are measured. See [6] for more details.

We remark that several measures for measuring linkage disequilibrium were proposed, including Pearson's correlation, Lewontin's *D′*, frequency difference and Yule's *Q*. Our proposal works for all these measures. However, we applied our method to Lewontin's *D′ *measure in the experimentation because of space limitations. Additional details related to these measurements are explained in an earlier report of the literature [6].

## Problem settings and threat model

### Problem settings

For our secure outsourcing of GWAS, we consider three stakeholders, *data contributors*, *researchers*, and *the cloud*. The data contributors (e.g. hospitals, research institutes or subjects) contribute private genomic or clinical data to the cloud. A researcher is an entity that wishes to conduct a GWAS. The cloud is an untrusted entity that includes researchers and data contributors with computational resources.

We assume that genotype/phenotype data of one subject can be contributed from different contributors. In other words, datasets *D^g ^*and *D^p ^*can be horizontally or vertically partitioned and can receive contributions from different contributors. Additionally, we assume that all subjects are identified with obfuscated IDs so that the cloud can correctly merge contributed data from two or more sources.

Given the contributed datasets *D^g ^*and *D^p^*, the protocol proceeds as follows. 1) The cloud computes sufficient statistics with *D^g ^*and *D^p^*, although it knows nothing about the contributed data and sends the resulting sufficient statistics to the researcher. 2) The researcher first reconstructs a frequency table from the sufficient statistics and then conducts GWAS.

### Threat model

The goal of our system is to ensure that 1) the cloud server cannot learn anything about the private data contributed by data contributors beyond the public information, such as the total number of subjects; 2) the researcher cannot learn beyond what is revealed by the frequency table. Even in the case in which the cloud server colludes with some contributors, they still have no means to learn anything about the data contributed by other contributors except the final results.

**Figure 1:**
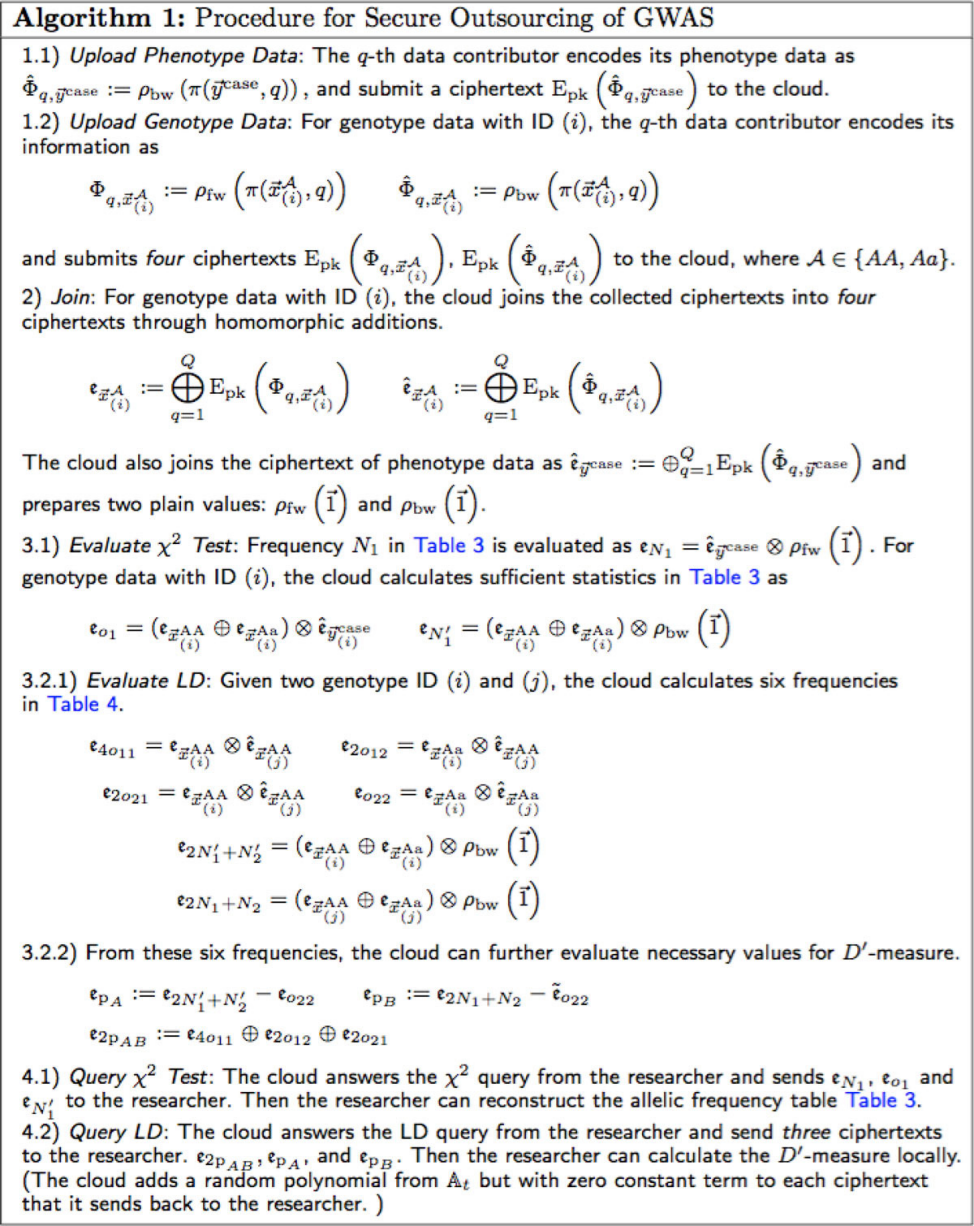
**Protocol of secure outsourcing of *χ*^2 ^test & linkage disequilibrium**.

In our setting, we assume that the cloud servers do not behave maliciously. However, the cloud server has motivation to learn some information related to the private data contributed by data contributors. This assumption naturally holds when the cloud server wishes to maintain a good reputation of their services. To avoid a man-in-the-middle attack, we assume that the key setup works correctly and that all data contributors obtain the correct encryption key from the analyst which can be enforced with appropriate use of Certificate Authorities. The Figure 1 to be described in the following section is thus designed to be secure against an honest-but-curious cloud server. Additional assumptions that must be made are the following.

1) The cloud server is not in collusion with the researcher to disclose private data contributed by data contributors. 2) Existence of a secure channel between data contributors and the cloud, e.g. SSH.

## Methods

Before description of our protocol, we first introduce a homomorphic encryption and packing technique used as building blocks of our protocol.

### Building block I: homomorphic encryption

Homomorphic encryption is a cryptosystem that allows performance of arithmetic operations of ciphertexts without decryption.

We detail a homomorphic encryption scheme based on ring-Learning with Errors (RLWE) assumption [7]. Let *n *be the lattice dimension of the scheme, where *n *is given as an integer of 2-power. Then, the message space of the scheme is given as a polynomial ring $A_{t}:{\mathbb{Z}}_{t}\left[x\right]/\left(x^{n}+1\right)$, where *t *is a prime number. Simply, we identify $A_{t}$ with the set of integer polynomials of degree up to *n *− 1 reduced modulo *t*. Moreover, we identify modulo *t *in the interval (−*t*/2, *t*/2].

For our implementation, we used HElib [8], which is an implementation of the Brakerski-Gentry-Vaikuntanathan (BGV) scheme proposed in [2]. The BGV's scheme is a public-key cryptosystem that supports homomorphic operations. Pre-suming that *m*_1_, *m*_2 _∈ $A_{t}$ are two plain polynomials and E_pk _(*m*_1_), *then *E_pk _(*m*_2_) are the corresponding ciphertexts encrypted by BGV's scheme under an encryption key pk. The BGV's scheme supports both homomorphic addition and multiplication:

Dsk(Epk(m1)⊕Epk(m2))≡m1+m2 mod(xn+1,t)Dsk(Epk(m1)⊗Epk(m2))≡m1×m2 mod(xn+1,t)Dsk(Epk(m1)⊕c)≡m1+c mod(xn+1,t)Dsk(Epk(m1)⊗c)≡m1×c mod(xn+1,t),

where *c *∈ $A_{t}$ and D_sk_(·) is the decryption function using the corresponding decryption key sk. It is noteworthy that homomorphic multiplication costs much more time than a homomorphic addition does in terms of magnitude.

We remark that the BGV's scheme supports the evaluation of circuits that are not deeper than a pre-defined level *L*. In other words, *L *denotes the maximal depth of evaluable circuits. The scheme security was analyzed intensively by Gentry et al. in [9]. We omit details of the security analysis and state their results below. The following equation describes the lattice dimension *n *that is necessary to evaluate deep-*L *circuits correctly with guarantee of *κ*-bits security,

$$n>\frac{\left(L\left(\text{log}\;n+23\right)-8.5\right)\;\left(\kappa +110\right)}{7.2}.$$

### Building block II: packing technique

The BGV encryption scheme takes *polynomials *as plaintexts. An integer vector is transformed into a polynomial form. Then the encryption function takes as input the polynomial and outputs a ciphertext, which also forms a polynomial [10,11]. These techniques are called packing techniques.

Transformations introduced by Yasuda et al. [10] were designed originally for secure Hamming distance evaluation of binary vectors. We introduce their method and designate the method as *forward *and *backward *packing. Letting $A_{t}$ be the given polynomial ring (with parameters *n*, *t*), and presuming that $\vec{u}$ and $\vec{v}$ are integer vectors with length *ℓ*, then forward packing *ρ*fw (·) and backward packing *ρ*bw (·) are defined respectively as

(1) $$\rho_{\text{fw}}\left(\vec{u}\right):=\overset{\ell -1}{\underset{i=0}{\sum }}u_{i}x^{i},\;\;\rho_{\text{bw}}\left(\vec{v}\right):=-\overset{\ell -1}{\underset{j=0}{\sum }}v_{j}x^{n-j}.$$

In the equations above, *u_i _*is the *i*-th element of $\vec{u}$;*u_j _*is the *j*-th element of $\vec{v}$. It is readily apparent that if *v_i_*, *u_i _*∈ (−*t*/2, *t*/2] for 0 ≤ *i *<*ℓ *and *ℓ *≤ *n*, then *ρ*fw and *ρ*bw respectively transform vectors $\vec{u}$ and $\vec{v}$ into elements of the ring $A_{t}$.

One benefit of this transformation is that homomorphic multiplication of the ciphertexts with this packing engenders a scalar product $\vec{u}\cdot \vec{v}$.

(2) $$\begin{array}{c} {\text{E}}_{\text{pk}}\left(\rho_{\text{fw}}\left(\vec{u}\right)\right)⊗{\text{E}}_{\text{pk}}\left(\rho_{\text{bw}}\left(\vec{v}\right)\right)\\ ={\text{E}}_{\text{pk}}\left(\overset{\ell -1}{\underset{i=0}{\sum }}u_{i}x^{i}\times \left(-\overset{\ell -1}{\underset{j=0}{\sum }}v_{j}x^{n-j}\right)\right)={\text{E}}_{\text{pk}}\left(-\overset{\ell -1}{\underset{i=0}{\sum }}\overset{\ell -1}{\underset{j=0}{\sum }}u_{i}v_{j}x^{n+i-j}\right)\\ \text{ = }{\text{E}}_{\text{pk}}\left(\overset{\ell -1}{\underset{i=0}{\sum }}u_{i}v_{j}x^{0}+\overset{\ell -1}{\underset{i=0}{\sum }}\overset{j+h<\ell }{\underset{j=0}{\sum }}u_{h+j}v_{j}x^{h}-\overset{\ell -1}{\underset{k=1}{\sum }}\overset{j+k<\ell }{\underset{j=0}{\sum }}u_{i}v_{j+k}x^{n-k}\right). \end{array}$$

The scalar product between vectors $\vec{u}$ and $\vec{v}$ is obtained from the constant term of Equation 2. The remaining 2*ℓ *− 2 terms are unconcerned.

Equation 2 allows evaluation of a scalar product between two length-*ℓ *encrypted vectors only by a single homomorphic multiplication. The correctness of this evaluation is presented in Theorem 1.

**Theorem 1 ***Let n be lattice dimension and t be prime modulo. Let *$\vec{u}$*and *$\vec{v}$*denote length-ℓ vectors. Then, the constant term of the decryption *$D_{sk}\left(e_{u}⊗{\hat{e}}_{v}\right)$, *where *$e_{u}:=E_{pk}\left(\rho_{fw}\left(\vec{u}\right)\right)$*and *${\hat{e}}_{v}:=E_{pk}\left(\rho_{bw}\left(\vec{v}\right)\right)$, *gives the scalar product *$\left\langle \vec{u},\vec{v}\right\rangle$*if (1) u_i_*, *v_i _*∈ (−*t*/2, *t*/2] *for *0 ≤ *i*, *j *<*ℓ; (2) ℓ *≤ *n; (3) *$\left\langle \vec{u},\vec{v}\right\rangle \in \left(-t\text{/}2,\;t\text{/}2\right]$.

The proof was obtained immediately from the derivation of Equation 2 and so is omitted here.

### Proposed secure outsourcing of GWAS

Recall that our goal is to outsource the evaluation of frequency tables efficiently while maintaining the genotype/phenotype data private to the cloud servers. We present an encoding scheme for genotype/phenotype data. Particularly, with this encoding, we can securely evaluate a frequency table through scalar products by the technique introduced into the previous section. We present a protocol for secure outsourcing GWAS in the last part of this section. The detail of the protocol is described in Figure 1.

#### Data encoding

Let *A *and *a *be the alleles of the biallelic locus. Consequently, the genomic data at the locus is either *AA*, *Aa*, or *aa*. We represent each row of the genomic dataset *D^g ^*as two integer vectors ${\vec{x}}^{AA}$, ${\vec{x}}^{Aa}$. Here, $x_{i}^{AA}$, the *i*-th element of ${\vec{x}}^{AA}$, represents the frequency of genotype *AA *at the marker locus: $x_{i}^{AA}=2$ for *AA *and $x_{i}^{AA}=0$ for other genotypes. $x_{i}^{Aa}$ is similar to $x_{i}^{AA}$ except that $x_{i}^{Aa}=1$ for *Aa*.

We presume that the disease status of each subject is represented by a binary variable, then "disease" is represented by 1 (case); "non-disease" is represented by 0 (control). The phenotype dataset D*^p ^*for all subjects is therefore represented by a binary vector ${\vec{y}}^{case}$.

Presume in addition to the following that dataset *D^g ^*consists of *N *SNPs with *M *subjects. *Q *data contributors are involved in the procedure. Therefore, they separately hold the phenotype vector ${\vec{y}}^{\text{case}}$ and 2*N *genotype vectors ${\vec{x}}_{\left(i\right)}^{\text{AA}}$ and ${\vec{x}}_{\left(i\right)}^{\text{Aa}}$, where (*i*) is the ID of the genotype data. Let *π *: {0, 1, 2}*^M ^*× {1, 2, ⋯, *Q*} ↦ {0, 1, 2}*^M ^*be an assignment function that represents the partition of genotype/phenotype held by the *q*-th data contributor. For example, the vertical partition of a vector $\vec{x}$ for the *q*-th data contributor is represented as shown below.

$$\pi {\left(\vec{x},q\right)}_{j}=\left\{\begin{array}{c} x_{j}\;\text{if}\;q\text{ - th}\;\text{data}\;\text{contributor}\;\text{holds}\;\text{the}\;j\text{ - th}\;\text{element}\;\text{of}\;\vec{x}\\ 0\;\;\;\text{o}.\text{w}. \end{array}\right).$$

We assume that each element of vectors is contributed from only one data contributor, i.e. $\sum_{q}\;\pi {\left(\vec{x},q\right)}_{j}=x_{j}$ holds for every *j*. For simplicity, we view $\pi \left(\vec{x},q\right)$ as a polynomial whose *j*-th coefficient has value $\pi {\left(\vec{x},q\right)}_{j}$.

We use this data encoding in Step 1.1 and Step 1.2 in Figure 1.

#### Evaluate the allelic frequency table

With the encoding described, we evaluate Table 3 through *scalar products *of the representing vectors. More specifically, frequencies *o*_1_, $N_{2}^{\prime }$, and *N*_1 _in Table 3 are evaluated respectively through three scalar products as

$$o_{1}=\left\langle {\vec{x}}^{AA}+{\vec{x}}^{Aa},{\vec{y}}^{case}\right\rangle ,N_{1}^{\prime }=\left\langle {\vec{x}}^{AA}+{\vec{x}}^{Aa},\vec{1}\right\rangle ,N_{1}=\left\langle {\vec{y}}^{case},\vec{1}\right\rangle ,$$

Where $\vec{1}$ is a vector of which the elements are 1. Because Table 3 is freedom-1 and the number of objects *M *is assumed to be known, whole Table 3 can be reconstructed with values *o*_1_, $N_{1}^{\prime }$ and *N*_1_. Therefore, three homomorphic multiplications are needed here. Step 3.1 of Figure 1 shows that the three scalar products can be evaluated with homomorphic multiplication.

#### Evaluate the genotype frequency table

Similarly, we compute the genotype frequency table described by Table 4 with two markers by scalar products of the represented vectors as well. In particular, to calculate a *D′*-measure for the LD, the following six scalar products are needed.

$$\begin{array}{c} 4\cdot o_{11}=\left\langle {\vec{x}}^{AA},{\vec{x}}^{BB}\right\rangle ,\;\;2\cdot o_{12}=\left\langle {\vec{x}}^{Aa},{\vec{x}}^{BB}\right\rangle ,\\ 2\cdot o_{21}=\left\langle {\vec{x}}^{AA},{\vec{x}}^{Bb}\right\rangle ,\;\;o_{22}=\left\langle {\vec{x}}^{Aa},{\vec{x}}^{Bb}\right\rangle ,\\ 2{N^{\prime }}_{1}+{N^{\prime }}_{2}=\left\langle {\vec{x}}^{AA}+{\vec{x}}^{Aa},\vec{1}\right\rangle ,\;\;2N_{1}+N_{2}=\left\langle {\vec{x}}^{BB}+{\vec{x}}^{Bb},\vec{1}\right\rangle . \end{array}$$

Step 3.2.1 of Figure 1 shows that the six scalar products can be computed with homomorphic multiplication as well.

#### Secure outsourcing GWAS protocol

The procedure of secure outsourcing GWAS is shown in Figure 1. Recall that the evaluation of scalar product in Equation 2 requires a forward-packed vector and a backward-packed vector. Consequently, at Step 1.2, data contributors upload four copies for one genotype data in the form of the forward-packed and backward-packed vectors. The cloud aggregates the collected ciphertexts at Step 2, which only involves homomorphic additions. Then the cloud computes the allelic frequency table and the genotype frequency table respectively at Step 3.1 and 3.2.

## Results

We benchmarked the computational costs of our method and compared it with a method proposed by Lauter et al. in [5], in which a genetic data point and a clinical data point are encoded respectively into three bits and two bits. All experiments were conducted on computers with a 2.60 GHz CPU (Xeon; Intel Corp.) and 32 GB RAM. We measured the computation time separately for Step 1.1 and 1.2 as the preparation time and for Steps 3.1 and 3.2 as the evaluation time. Details of the experiment settings are presented following. 1) An artificial dataset includes 1.0 × 10^4 ^subjects. 2) *Q *= 5 data contributors are sharing same quantity of data points. 3) We used 8 threads for computation in parallel. 4) Parameters of the encryption scheme were set as *n *= 8192, *t *= 640007, and *L *= 6.

### Performance of homomorphic encryption and implementation hints

The implementation of Lauter et al. was done on an algebraic computation system, Magma, whereas our implementation was developed on native codes. To compare our method with their method fairly, we measured the computation time of operations in HElib and re-estimated the computation time method of Lauter et al. Table 5 shows the computation time of the operations of homomorphic encryption scheme. Values are the mean of 1000 runs of each operation with 8-threads. We used parameter *n *= 8192, which is not sufficiently large to conduct more than 8192 subjects. Indeed, we partitioned vectors into smaller parts and encrypted each part as a ciphertext. In doing so, we were able to conduct a large-scale dataset while maintaining smaller *n*. We remark that as the number of the partition increases, more communication time must be used during the upload phase.

**Table 5:** Timing of fully homomorphic scheme with parameters *n *= 8192, *t *= 640007, *L *= 6.

Operation	Encrypt	Mult	Add	Add with Plaintext
Time (ms)	3.08	7.57	0.032	0.789

### Artificial genotype & phenotype dataset

**Figure 2:**
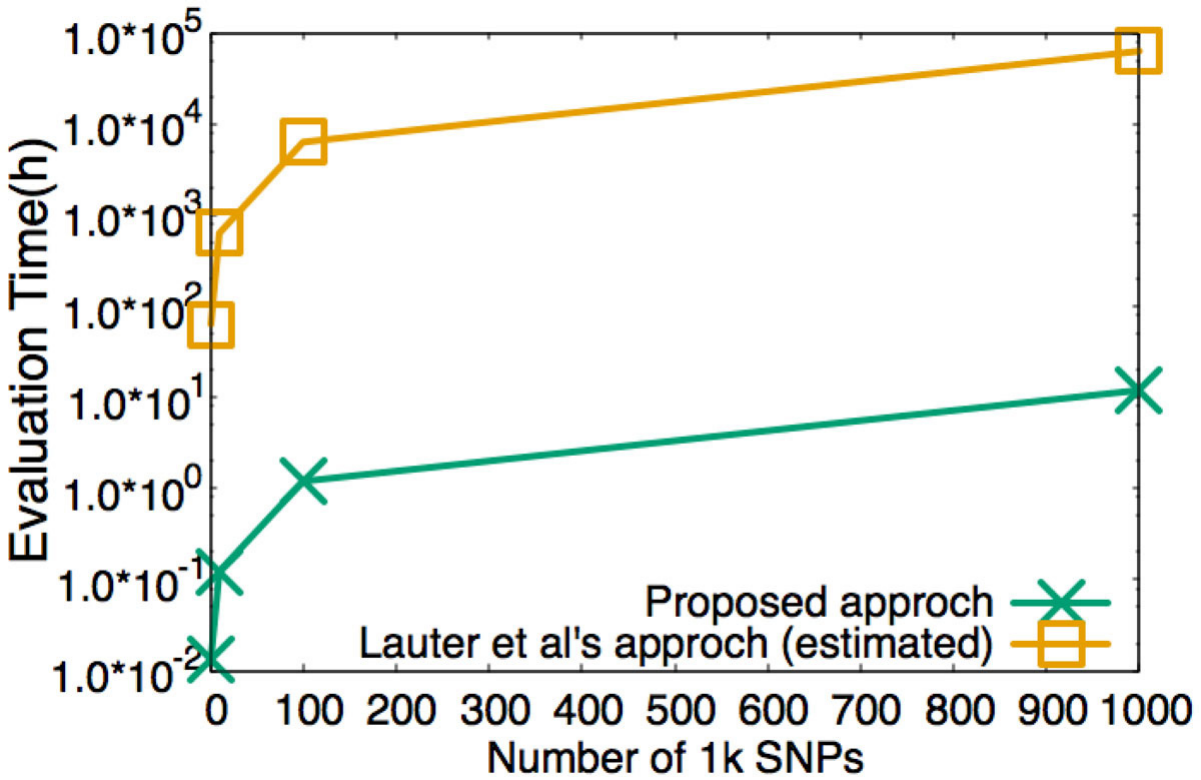
**Benchmark for outsourcing *χ*^2 ^test with 1.0 × 10^4 ^subjects**.

We benchmarked our proposed protocol of evaluating *χ*^2 ^test on an artificial dataset that contains 1.0 × 10^4 ^subjects. The results are presented in Figure 2. The number of the total SNPs was varied from 1.0 × 10^3 ^to 1.0 × 10^6^. At Step 3.1 of the Figure 1, only three homomorphic multiplications are necessary to evaluate a *χ*^2 ^test statistics. Recalling that parameter *n *= 8192, one can thereby maximally pack genotype/phenotype data of 8192 subjects into a single ciphertext. Consequently, to conduct the experiment with 1.0 × 10^4 ^subjects, we partitioned a vector into two parts having equal length. Figure 2 depicts the performance of our proposed method and the estimated computation time of the method of Lauter et al. [5]. As shown in Figure 2, for evaluation of *χ*^2 ^test statistics of 1.0 × 10^6 ^SNPs with 1.0 × 10^4 ^subjects, our method took about 12 hours (about 43 ms per test).

**Figure 3:**
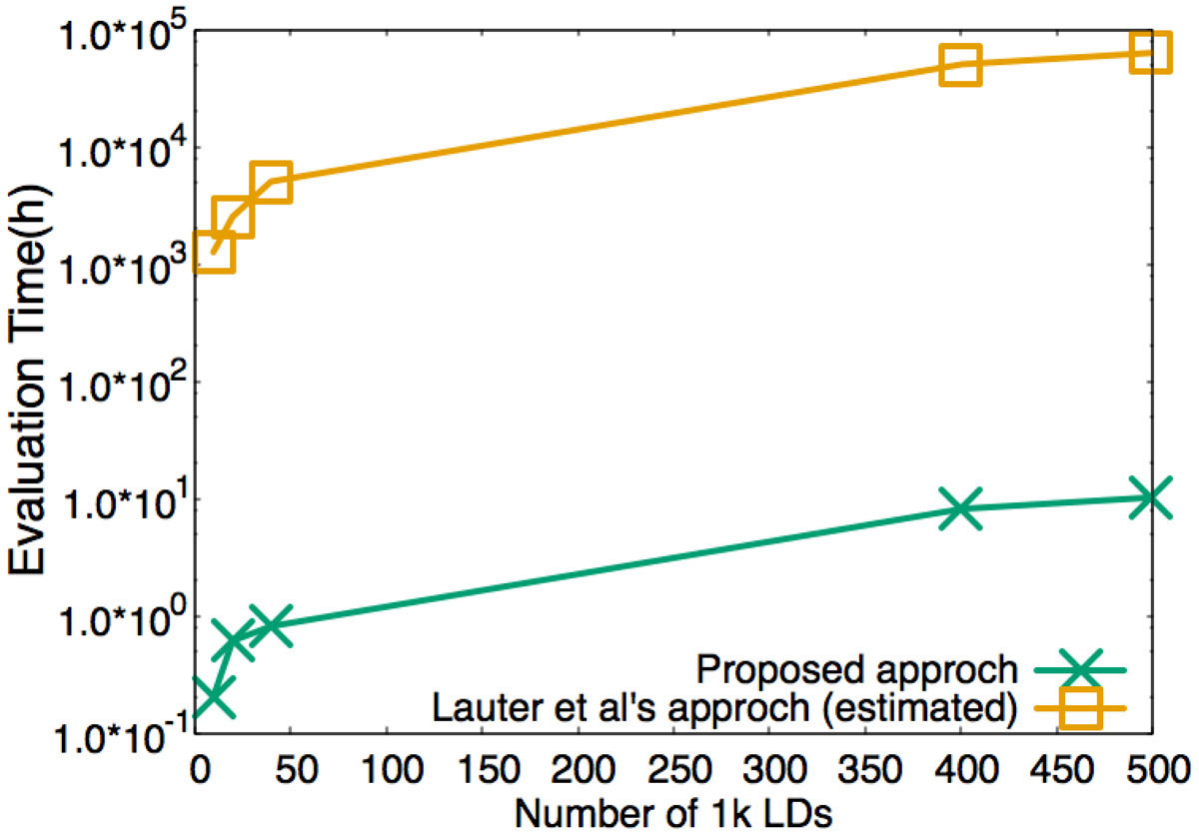
**Benchmark for outsourcing LD with 1.0 × 10^4 ^subjects**.

The benchmark of the evaluation of LD is presented in Figure 3. In this experiment, we considered a smaller synthetic data containing 1.0 × 10^3 ^SNPs of 1.0 × 10^4 ^subjects. The number of LD to be evaluated with *p *SNPs is *p*(*p *− 1)/2. We therefore evaluated about 5.0 × 10^5 ^LDs in this experiment. With this settings, our method costs less than 11 hours (about 80 ms per LD).

## Conclusions

From Figure 2 and 2 we can see that Lauter et al's cryptographic solution [5] might take about 2000 days to conduct the evaluation of *χ*^2 ^test of one million SNPs and takes about 2600 days to conduct the evaluation of half million of linkage disequilibrium. At the meantime, it respectively took our approach about 12 hours and 11 hours to conduct the same computation. We conclude that with the appropriate encoding and packing technique, secure outsourcing of GWAS using FHE can be practical.

## Related work

Studies of privacy-preserving data processing in GWAS involve different techniques. Kamm et al. proposed a secret sharing-based method in [12], by which private information is divided into several parts and is transferred to at least three collusion-free servers. All servers share the workload equally. The final result is aggregated from the output of each server. Computation based on secret-sharing requires multiple rounds of communication between servers; the computation is secret as long as no two servers collude. Because our outsourcing approach executes the whole computation with single cloud servers, computational environments employed for the computation are different.

A cryptographic solution was proposed recently from the work of Lauter et al. [5]. They constructed a method for computation on encrypted genomic data using a cryptosystem that is similar to BGV's scheme. Each genetic datum is encoded into three ciphertexts, which can cause inefficiency in both time and space. Our previous work [13] proposed a specified approach for secure outsourcing *χ*^2 ^test. In this manuscript we propose a more general approach for secure outsourcing of *χ*^2 ^test, HWE and LD etc.

An orthogonal method to ours is differential privacy [14]. With perturbation noise, differential privacy ensures that distribution of the output is insensitive to any data contributor's record, making it impossible to infer data from the obfuscated output. In our case, we can incorporate the perturbation noise in the query phase. Therefore, differential privacy can enforce the privacy properties of our protocol.

## Competing interests

The authors declare that they have no competing interests related to this study.

## Authors' contributions

Wen-jie Lu and Jun Sakuma designed the algorithm and drafted the majority of the manuscript. Wen-jie Lu conducted the experiments. Yoshiji Yamada gave useful comments on bio-information and provided genotype and phenotype data.

## Declarations

Publication funding for this supplement was supported by iDASH U54HL108460, iDASH linked R01HG007078 (Indiana University), NHGRI K99HG008175 and NLM R00LM011392.
